# Molecular Determinants of Malignant Brain Cancers: From Intracellular Alterations to Invasion Mediated by Extracellular Vesicles

**DOI:** 10.3390/ijms18122774

**Published:** 2017-12-20

**Authors:** Gabriella Schiera, Carlo Maria Di Liegro, Italia Di Liegro

**Affiliations:** 1 Department of Biological Chemical and Pharmaceutical Sciences and Technologies (STEBICEF), University of Palermo (UNIPA), I-90128 Palermo, Italy; gabriella.schiera@unipa.it (G.S.); carlomaria.diliegro@unipa.it (C.M.D.L.); 2 Department of Experimental Biomedicine and Clinical Neurosciences (BIONEC), University of Palermo, I-90127 Palermo, Italy

**Keywords:** glioma cells, brain cancer invasion, extracellular vesicles (EVs), ECM, extracellular RNAs

## Abstract

Malignant glioma cells invade the surrounding brain parenchyma, by migrating along the blood vessels, thus promoting cancer growth. The biological bases of these activities are grounded in profound alterations of the metabolism and the structural organization of the cells, which consequently acquire the ability to modify the surrounding microenvironment, by altering the extracellular matrix and affecting the properties of the other cells present in the brain, such as normal glial-, endothelial- and immune-cells. Most of the effects on the surrounding environment are probably exerted through the release of a variety of extracellular vesicles (EVs), which contain many different classes of molecules, from genetic material to defined species of lipids and enzymes. EV-associated molecules can be either released into the extracellular matrix (ECM) and/or transferred to neighboring cells: as a consequence, both deep modifications of the recipient cell phenotype and digestion of ECM components are obtained, thus causing cancer propagation, as well as a general brain dysfunction. In this review, we first analyze the main intracellular and extracellular transformations required for glioma cell invasion into the brain parenchyma; then we discuss how these events may be attributed, at least in part, to EVs that, like the pawns of a dramatic chess game with cancer, open the way to the tumor cells themselves.

## 1. Introduction

The cancers of the Central Nervous System (CNS) are extremely complex and heterogeneous [1,2,3]. The most common of them derive from glial cells and are called gliomas, further subdivided into astrocytomas, oligodendrogliomas, ependymomas and glioastrocytomas [4,5]. Among gliomas, the most aggressive (i.e., glioblastoma multiforme, GBM, grade IV) are histologically characterized by a high mitotic index, accompanied by hypoxia, necrosis and microvascular proliferation [6]. Actually, a high degree of inter-observer variation of the histopathological diagnosis has been reported in clinical trials on gliomas, thus suggesting the need for more precise analyses of the tumors [7]. A recent classification of gliomas was based on mutations present in specific genes [8,9,10]; in particular, an integrated genomic analysis identified clinically relevant subtypes of glioblastoma, characterized by abnormalities in PDGFRA, IDH1, EGFR and NF1 [11]. According to these observations, glioblastomas have been subdivided into: (i) proneural (PN), characterized by mutations in the genes encoding isocitrate dehydrogenase genes 1 and 2 (*IDH1/2*) (mutations frequently found in secondary glioblastomas), platelet derived growth factor receptor α (PDGFRα) and *TP53* (which encodes p53 oncosuppressor protein); (ii) neural (N), which express high levels of neuronal markers, such as neurofilament light polypeptide (NEFL) and the synaptic protein synaptotagmin (SYT1); (iii) classical (C), which frequently show amplification of the gene encoding the epidermal growth factor receptor (EGFR) and (iv) mesenchymal (MES), in which mutations in the genes encoding neurofibromin 1 (NF1), a negative regulator of Ras signaling pathway, phosphatase and tensin homolog (PTEN) and TP53 have been reported. Among the four subtypes, the most aggressive are the MES glioblastomas [11,12,13]. More recently, however, it became clear that heterogeneity is even higher than previously expected; data based, indeed, on single cell RNA sequencing suggest that primary glioblastomas differ even at the single cell level [14] and that the tumor, as a whole, is a sort of “ecosystem”, made up of cells that show a variety of phenotypes and also of genotypes and even differ at the epigenetic level [15].

Actually, more and more biomarkers continue to be identified in patients [16], more or less specific for one or more of GBM subtypes; some biomarkers (e.g., the mitotic spindle checkpoint molecule BUB1B) have been even suggested to be relevant for the prognosis, regardless of tumor subtype [17].

In spite of the progresses done in understanding their biology and in finding out specific prognostic markers, GBMs are still fatal [18]. The therapy, based on surgery (as extensive as possible), followed by radiotherapy and chemotherapy directed to reduce cell growth (e.g., Temozolomide) [19] and angiogenesis (e.g., Bevacizumab) [20] is indeed not yet sufficient to reach all the infiltrating cells and less than 10% of patients survive for more than three years [6].

We thus need a still better knowledge of GBM biological properties and more powerful methods for their as early as possible diagnosis.

## 2. Cellular and Molecular Bases of Glioma Growth and Invasion

As mentioned above, one central property of GBM is its heterogeneity, which is due to the presence, in the tumor, of cells with different degrees of differentiation, among which glioblastoma stem cells (GSCs).

GSCs are supposed to be well adaptable to hypoxia and capable of self-renewal; these GSC properties are also believed to be responsible for therapeutic resistance of cancer and for its recurrence [21,22].

Another important feature of gliomas is their low or even absent metastatic invasion outside the brain. It is not clear whether this behavior is due to inability of glioma cells to cross the blood-brain barrier (BBB), or to the need of a specific environment for growth, only found inside the brain [6]. Although they do not cross the basal membrane of brain capillaries, cancer cells can invade the brain parenchyma, moving along the vessels in small groups (model of the guerrilla war) [23]. In addition, a sequential switching of cells between proliferation and invasion has been reported during tumor progression. In other words, it seems that proliferation and migration are temporally, mutually exclusive phenotypes [24,25].

In order to invade the brain parenchyma, glioma cells must modify their own interactions with the ECM and the ECM itself, which in the brain (see below) has a peculiar composition [26]. Moreover, the rapid proliferation of the malignant cells per se has a metabolic effect on the microenvironment, which is rapidly deprived of glucose and oxygen, becoming acidic and hypoxic [6]. These modifications are part of the so called “epithelial-mesenchymal transition” (EMT) (see Section 2.2), which, although its role in glioma is still controversial, seems to be determinant for the degree of malignancy [27]. In addition, movement of cells throughout the brain tissue requires cell shape changes and protrusion of invadopodia, probably based on both modifications of the cytoskeleton [28,29] and movements of ions [30,31] and water [32,33,34] between the two sides of the plasma membrane. In this Section, we discuss these molecular modifications, while in Section 3 we will discuss possible involvement of EVs in them.

### 2.1. The Extracellular Matrix (ECM)

Extracellular matrix (ECM) is an intricate network of macromolecules, connected both among them and to the surface of the cells; it is now widely accepted to represent not only an inert scaffold, able to stabilize the physical structure of tissues but also the substrate on which tissue cells can adhere, migrate, proliferate and differentiate. ECM also binds molecules such as growth factors and plays an active and complex role in controlling cell behavior in morphogenesis, pathophysiology, tumor invasion and metastasis. The set of proteins that make up the ECM, the factors that remodel it and the proteins associated with it have been termed “matrisoma” [35]. 

The existence of an ECM in the central nervous system (CNS) has been known since 1971 [36]. CNS ECM is composed of several glycosaminoglycans, proteoglycans and their binding partners, link proteins and tenascins [37]. 

The most abundant component is hyaluronan (HA), associated with a number of proteins, organized into a scaffold. Chondroitin sulfate proteoglycans (CSPGs) are, however, also abundant in the brain and include lecticans such as aggrecan, brevican, neurocan and versican, phosphacan (a tyrosine phosphatase) and small leucine-rich proteoglycans [6].

ECM is a highly dynamic structure whose remodeling is regulated by ADAMT (a disintegrin and metalloproteinase with thrombospondin motifs) family of enzymes, as well as by matrix metalloproteases (MMPs) [37].

At the base of invasion and progression of glial tumors there is a continuous interaction between neoplastic cells and ECM. The sequence of events involves: (i) synthesis of extracellular matrix components by tumor and mesenchymal cells, (ii) release of enzymes which degrade specific ECM molecules, thus remodeling the interstitial space and (iii) de novo expression of adhesion molecules (receptors for matrix) on the surface of glioma cells, which specifically recognize and adhere to the components of the ECM itself. Moreover, it has been found that rigidity of the ECM regulates the motility of glioma cells as well; in particular, a stiffer ECM will be invaded by glioma cell more easily [26,38].

A variety of ECM components have been found to be either up- or down-regulated in brain tumors and have been therefore considered as possible key molecules in the mechanism of invasion of malignant glial tumors; some of them are listed in Table 1. As discussed below, remodeling of ECM can be mediated by the release of extracellular vesicles containing both components of the ECM itself and enzymes that degrade it, as well as by deregulated production of both coding and noncoding RNAs which target mRNAs encoding proteins involved in ECM structure and function.

As reported in the last column of Table 1, many attempts have been made to specifically target ECM molecules involved in glioma cell invasiveness. These methods can be collectively grouped into two categories: (i) methods based on the use of natural/synthetic compounds that can directly function as inhibitors of ECM enzymes and (ii) methods based on knocking down genes, more frequently encoding transcription factors, or components of transduction pathways, which are known to be altered in gliomas. In both cases, a significant inhibition of the activity of different classes of matrix enzymes has been obtained and this resulted, at least in vitro, in reduction of the ability of the cells to move. However, a main problem still encountered is the difficulty to obtain high drug penetration into the brain parenchyma, the structure of which is highly compact, mainly due to the abundance of hyaluronic acid. For this reason, an interesting approach used on model mice has been intratumoral injection of a conditionally replicating adenovirus expressing soluble hyaluronidase (ICOVIR17). The method successfully allowed viral spreading and might offer an efficient way to ensure better penetration throughout the brain parenchyma of different kinds of drug-loaded nanoparticles [54].

### 2.2. The Cytoskeleton

One of the critical morphological changes that the cell undergoes during neoplastic transformation is the transition from a well differentiated phenotype, which ensures regulated interactions with the neighboring cells and with the ECM, to a phenotype capable of motility and invasiveness. A process of this kind can allow polarized epithelial cell to detach from the basement membrane and to assume a mesenchymal phenotype, endowed with new properties, such as enhanced migratory potential, high resistance to apoptosis and invasiveness. Cancers of epithelial origin actually seem to undergo this transformation, known as epithelial-mesenchymal transition (EMT). EMT, also important during physiologic repair of injured tissues, as well as in embryogenesis [76], occurs through an orchestrated series of sequential events: (i) cell–cell interactions and extracellular matrix-cell interactions are altered, (ii) the cytoskeleton reorganizes in order to allow migration through the ECM and epithelial cells are released into the surrounding tissue and (iii) a new transcriptional program is activated, which allows tumor cells to maintain an invasive mesenchymal phenotype, which can give rise to metastases [27].

Although the cells from which gliomas derive are not typical polarized epithelial cells, with an apical side and a basal side bound to a basement membrane, the concept of EMT is still useful to describe the ability of these cells to acquire a migratory phenotype. As mentioned above, even glioblastoma cells (the most malignant) only rarely form metastases outside the brain; they are however, able to invade the brain parenchyma, by moving along the brain capillaries.

One of the most relevant phenomena in EMT is the rearrangement of cytoskeletal structures, normally fundamental for maintaining cellular shape. The cytoskeleton is actually a dynamic structure, consisting of three different components: microtubules, actin filaments and intermediate filaments. Microtubules were considered to be the main drivers of changes related to increased cell motility in cancer. Recently, the importance of microtubule proteins also on changes in actin cytoskeleton, mediated by activation of Rho GTPase, has been seen. In GBM, Class III β-tubulin and α-tubulin are overexpressed; these two proteins not only have different, anomalous, subcellular sorting but also interact with each other forming complexes, which induce cytoskeleton rearrangements, resulting in increased motility [77,78,79]. Actually, the cells expressing (βIII)-tubulin form a small group with the properties of cancer stem cells, which are localized in ischemic necrotic areas [78]. This observation led to the hypothesis that this protein can provide protection from oxidative stress and hypoxia, also because it lacks cys239 and may allow (βIII)-tubulin to be assembled into microtubules also in the presence of free radicals [80].

The mentioned cross-talk between microtubules and actin cytoskeleton relies, at least in part, on stathmin (STMN1), a phosphoprotein with a key role in cell motility and migration, which is involved in the RhoA/ROCK signaling pathway. In glioblastoma, STMN1 is a target of microRNA-9 [81].

At the molecular level, many of the above cited phenomena are largely driven by the recruitment of small monomeric members of the Rho GTPase family. Variations in the levels of these proteins or of Rho-associated, coiled-coil-containing protein kinase (ROCK) affect glioma cell migratory phenotype [82]. The Rho-family GTPases, such as Cdc42, Rac1 and RhoA, have been shown to influence invadopodia formation. Invadopodia are actin enriched protrusions, which also contain actin-binding proteins such as Arp2/3. Thanks to a number of multiple transmembrane- (e.g., MT1-MMPs) and secreted-proteins, these structures mediate proteolysis of ECM constituents, including fibronectin, laminins and collagens [83].

Rho proteins can be considered as molecular “switches”, whose functional state changes periodically from a guanosine diphosphate (GDP)-bound, “inactive” state, to a GTP-bound, “active” state. In the active form, Rho proteins are able to bind a wide range of effectors or target molecules, thus modulating various cellular activities. Activation of Rho proteins depends on specific regulators known as guanine nucleotide exchange factors (GEFs) [84], while, on the other hand, their inactivation is stimulated by RhoGAPs (RhoGTPase-activating proteins) [85].

The highly conserved RhoA, RhoB and RhoC proteins are frequently aberrantly expressed in human tumors [86]. RhoG, which stimulates lamellipodia formation, is often overexpressed in gliomas; it is also able to activate Rac1, with a further increase in cell migration [28]. Rac1, which promotes invasive glioma cell behavior, can be overexpressed too [29] and can be also activated downstream of signaling networks triggered by Ephrin-B3 ligand [87], EGFRvIII receptor [88], or PDGFRα receptor [89].

Another important cytoskeleton regulator is cofilin that can bind both monomeric and filamentous actin and is able to regulate polymerization/depolymerization processes [90,91]. Studies based on the use of specific, small interfering RNAs (siRNAs) demonstrated that blocking cofilin expression causes reduction of carcinoma cells invasion, while its overexpression increases the rate of cell migration in human glioblastoma cells [92]. Moreover, cofilin localization seems to be regulated by Na^+^/K^+^/2Cl^−^ co-transporter 1 (NKCC1), which probably hooks the protein to the plasma membrane [93].

Actually, also other actin-binding proteins have been reported to be overexpressed in glioma cells. For example, ezrin, which acts as a linker between the actin cytoskeleton and the plasma membrane, has been considered a biomarker of glioblastoma, where it is overexpressed respect to normal astrocytes [94]. In the same manner, fascin [95] and actinin-4 [96] are also overexpressed in GBM.

Actin-related protein 3 (Arp3) regulates actin polymerization, lamellipodia formation and cell migratory phenotype; it is also able to bind RAS Guanyl Releasing Protein 3 (RasGRP3), thus modulating its function. RasGRP3 is a protein with a Ras guanine nucleotide exchange factor (RasGEF) function, also implicated in proliferation and migration of glioma cells. Both Arp3 and RasGRP3 can be overexpressed in gliomas [97].

Beside microtubule and actin cytoskeleton alterations, also intermediate filaments (IF) have been found to undergo modifications in brain tumors. For example, nestin expression is related to a high glioma grade and with a poor prognosis for patients [98]. Similarly, synemin overexpression affects cell motility and can influence proliferation through the alpha serine/threonine-protein kinase (Akt) pathway [99]. Moreover, overexpression of vimentin and α-internexin appears to correlate with a negative clinical outcome [100].

GFAP (glial fibrillar acidic protein) is an astrocytic differentiation marker that also belongs to the family of intermediate filaments. This protein was found in high concentrations (>100 ng/L) in serum of patients with glioma, where its level correlated with the tumor volume [101,102]. In a very recent study, GFAP levels in the serum have been also studied during the follow-up of patients, who underwent surgery. It was found that all initially GFAP positive GBM patients showed decreased serum GFAP concentrations after surgery. However, although almost all the patients showed tumor progression or died, only a minimal GFAP increase was found and only in one patient, thus suggesting that GFAP is not predictive for tumor recurrence [103].

### 2.3. Transcription Factors

Several signal transduction systems are involved in the genesis of glioblastoma and many transcription factors have been reported, as well, to affect glioma cell ability to proliferate, escape apoptosis, migrate and invade neighboring tissues. For some of them, the amount of evidences underscoring their role in cell transformation and malignity promoting activity is really conspicuous.

One of the best characterized pathways involves β-catenin, the effector transcription factor of the WNT pathway; β-catenin regulates the transcription of different genes involved in cell proliferation and differentiation. In glioblastoma (GBM) and anaplastic astrocytomas, WNT signaling is misregulated and both β-catenin and the transcription factor 4 (TCF4) show abnormal expression levels [104]; in particular, increased nuclear amount of β-catenin are found in those patients presenting the mesenchymal type of GBM [105]. As mentioned, cancer growth and invasion are most probably due to the capacity of some cells in the tumor to conserve stemness characteristics [106], keeping high expression of Nanog, Oct4, Sox2 and c-Myc, all of which are TCF4/β-catenin targets [107]. Directly connected with β-catenin activity is the role of Forkhead box protein M1 (FoxM1), whose abnormal expression has been often found in glioma [108]. Some reports underscored the capacity of FoxM1, in GBM, to bind β-catenin in the cytoplasm and translocate it to the nucleus in a Wnt-independent fashion [109,110], increasing the expression level of *c-Myc* and *cyclin D1* genes [110], as well as of Myb-related protein B (MYBL2) [111]. FoxM1 might be in turn upregulated by high-mobility group AT-hook 2 (HMGA2), a protein that was demonstrated to be highly expressed in Grade II-IV gliomas, supporting GBM cell invasive behavior [112]. The activity of the Zinc finger E-box-binding homeobox 2 (ZEB2) protein, a factor highly expressed in GBM patients with fast tumor progression, also appears to be positively correlated to β-catenin expression [113], while the factor SRY-Box 7 (SOX7) could probably act as a repressor of the Wnt/β-catenin pathway [114]. 

Iperactivation of the nuclear factor (NF)-κB is commonly found in GBM as well [115] and it has been often associated with the mesenchymal phenotype [116]. In some GBM forms, NF-κB function has been correlated with iperactivation of epidermal-(EGFR) and platelet-derived-(PDGFR) growth factor receptors [117,118]; moreover, NF-κB was associated with mesenchymal phenotype acquisition in response to tumor necrosis factor (TNF) stimulation in cultures of GSC derived from GBM patient [119]. Actually, NF-κB has been reported to activate genes involved in mesenchymal transition, such as *Snail*, *ZEB1*, *ZEB2*, *Twist*, *MMP-2* and *MMP-9* [120] and, in general, genes encoding for proteins able to potentiate glioma cell ability to invade surrounding parenchyma, such as the TNF-like weak inducer of apoptosis (TWEAK-Fn14) [121]. It has been suggested that invasive behavior in glioblastoma could be enhanced by a cross-talk between glioma cells and the neighboring astrocytes, based upon NF-κB/RANKL signaling pathways [122]. 

Although activation of NF-κB seems a very frequent event in brain tumors, the pathway(s) leading to its activation are not yet completely understood. Recently, it was found, for example, which netrin-1, a protein probably involved in axon guidance during brain development, is highly expressed in glioma cells, in a tumor grade-dependent way; it was suggested that netrin-1 activates NF-κB in an Unc5 netrin receptor A-dependent route, resulting in increased c-Myc expression [123]. Another study suggested that NF-κB and JAK1-STAT3 pathways can be activated by CUE domain-containing protein 2 (CUED2), influencing glioma development [124]. One consequence of the activation of NF-κB but also of other transcription factors (e.g., AP-1 and Sp1), which are downstream to the mitogen-activated protein kinase (MAPK) and PI3/Akt pathways, is that they might enhance the expression of MMPs [125,126] and of some of the transporters related to glycolytic metabolism, such as GLUT3 [127].

Among the factors acting downstream to the PI3K/Akt signaling pathway, Snail seems to be expressed in direct correlation with GBM mesenchymal phenotype and tumor invasiveness [128], properties that Snail would promote by altering E-cadherin levels [129]. However, the regulation of Snail expression appears quite complex, because it is controlled by many different signaling molecules, including Wnt, TGFβ and HIF-1α [128]. 

While discussing the pathways that have been found altered in gliomas, it is to underline that, as reported in much detail in a later section, hypoxia has a central role in the acquisition of the stemness potential by GBM cells. HIF-1α, which is directly activated by hypoxia, was indeed shown to be necessary for GSCs maintenance [130]; once activated, it stimulates angiogenesis through upregulated expression of TGF-β, PDGF/PDGF-R and VEGF/VEGF-R [131], as well as the expression of stemness factors, such as cMet and CD133 [132]. Moreover, HIF-1 seems to regulate Twist, a factor involved in metastasis [133] and in escaping apoptosis in neuroblastoma [134]; in addition, it induces the expression of CXCR4, a factor previously reported as a mediator of invasiveness [135] and cell migration [136]. HIF-1 also controls metabolism: adaption to hypoxia includes indeed a switch to anaerobiosis and HIF-1 seems to be directly involved in the process by inducing pyruvate kinase M2, phosphoglycerate kinase and aldolase [137]. The hypoxic state stimulates also the expression of HIF-2α that, in turn, increases the levels of known reprogramming factors such as Oct-4, Nanog, Sox-2 and c-Myc [138]. 

All the signaling pathways described above somehow converge on the activation of transcription factors involved in EMT, such as ZEB1 [105], Twist [139], Snail [140] and Slug [141] and in MET [142]. Now, GBM is characterized by the aberrant co-expression of many genes involved in maintaining a pluripotent state and contrasting differentiation, such as *OCT4*, *Nanog*, *Sox2* [143,144]. In general, all those factors required for reprogramming cells, like POU3F2 (OCT7), Sox2, Sall2 and Olig2 are highly expressed in GBM and in more than 50% of cancers presenting high expression of these four transcription factors also CD133 is expressed [145]. Sox2 is nearly always overexpressed in human brain cancer biopsies [146] and, when expressed ectopically, confers to the receiving cells the ability to invade and migrate through the ECM [147]. As told about Snail, also Sox2 has a rather complex behavior: at least four different signaling pathways (i.e., TGF-β, SHH, EGFR and FGFR) have been indeed described as modulators of its expression [148]. For example, it has been demonstrated that Gli transcription factor, acting downstream in the Sonic hedgehog pathway, can stimulate both genes involved in maintenance of self-renewal capacity (e.g., *Sox2* and *Nanog*) and genes involved in EMT, like *Snail* [149]. In general terms, it seems that, in glioma cells, stemness and mesenchymal phenotype are closely linked: knockdown of genes directly involved in EMT, such *ZEB1*, also causes inhibition of stem cell regulators like Sox2 and Olig2 [150]. Interestingly, Singh and coll. [151] have recently demonstrated that Olig2 activity is regulated by phoshorylation: in particular, unphosphorylated Olig2 induces TGF-β2 pathway and Smad2 expression and increases ZEB1 expression. Moreover, a direct interaction between Olig2 and ZEB1 seems to exist and cause reciprocal stimulation, reinforcing the invasion capacity of glioma cells [151].

Finally, a direct correlation between CCAAT-enhancer binding protein (C/EBP) expression and tumor grade, as well as survival, has also been demonstrated in glioma patients [152]. It was indeed shown that C/EBP depletion enhanced the activity of genes involved in G0/G1 checkpoint and DNA damage response, leading to the inhibition of proliferation, thus demonstrating that C/EBP has a stimulatory effect on glioblastoma cell proliferation and survival, by directly controlling the cell cycle [153]. 

As we shall see below, alteration of one of more of these pathways can be caused by deregulation of the expression of specific miRNAs and/or lncRNAs, which target the mRNAs encoding transcription factors or other elements of the transduction routes leading to their activation/inhibition.

### 2.4. Ion and Water Channels 

Glioma cell invasion into the surrounding parenchyma requires, besides modifications of the cell shape and production of invadopodia, also adjustment of cell volume. In 1999, by using a Transwell migration system, Soroceanu and colleagues showed that blockade of glioma Cl(−) channels specifically inhibited glioma cell migration in a dose-dependent manner, thus suggesting that chloride channels can have an important role in cell invasiveness, presumably by facilitating acquisition of cell shape/volume more suitable for migration and penetration into the surrounding tissue [154]. More recently, by using quantitative three-dimensional multiphoton and confocal time-lapse microscopy, Watkins and Sontheimer analyzed glioma cell invasion in vivo and in vitro. They found that, actually, in all the conditions observed, invading cells showed a 30–35% reduction of volume [155]. Cell shrinking is due to reduction of cytoplasm and this process seems to depend on coordinated secretion of K^+^ and Cl^−^ ions and water. Ion channels involved in migration are mostly localized on invadopodia [30].

Taken together, these observations suggest that modifications of volume are critical for invasion and that they depend, at least in part, on ion fluxes and in turn on the expression of ion channels. It has been, for example, reported that CLC-3, one member of CLC voltage-gated chloride channel family, is upregulated in gliomas and correlates with a shorter survival of patients [31]. Similarly, the chloride intracellular channel 1 (CLIC1) is overexpressed in glioblastoma, with the highest expression in patients with worse prognosis [156]. Moreover, CLIC1 has been shown to exist also as a circulating protein, transported by extracellular vesicles [157]. 

Beside voltage-gated channels, recent studies have also suggested involvement of non-voltage-gated calcium channels (also called transient receptor potential channels: TRPC) in glioma cell proliferation, migration and invasion; interestingly, preclinical mouse models suggest that inhibition of TRPC channels have promising anti-cancer effects [158].

In human brain tumors, a reduced expression or mislocation of the Kir4.1, one member of the inwardly rectifying potassium channel family, has been also detected [159]. Interestingly, expression of this channel is regulated by the pro-invasive micro-RNA 5096 (miR-5096) [160].

**Figure 1:**
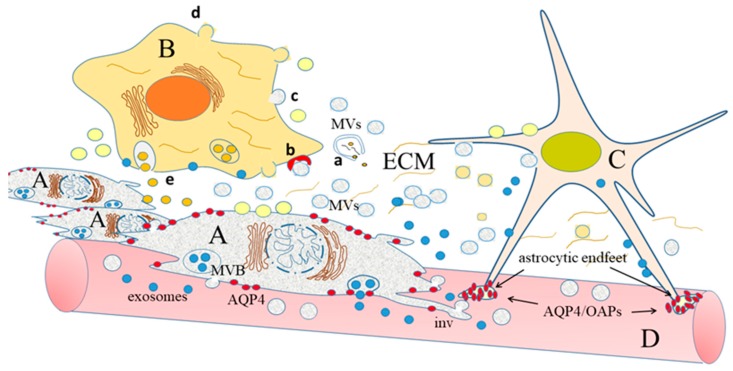
Cross-talk between glioma cells (**A**) and other cells (**B**,**C**), embedded in the extracellular matrix (ECM). The glioma cells have acquired the ability to move through the brain parenchyma, along the blood vessels (**D**), in small groups (guerrilla war) [23]; their invasiveness is mostly due to the extension of invadopodia (inv) and to the release of different kinds of extracellular vesicles: (i) membrane vesicles (MVs), light grey, which originate by directly budding from the plasma membrane and (ii) exosomes, blue, which are released after fusion with the plasma membrane of multivesicular bodies (MVB), components of the endosomal compartment. Both kinds of vesicles are equipped with different molecules (lipids, proteins and RNAs od different classes), which can be directly released into ECM if the vesicles break outside the cells (**a**). Alternatively, EVs can be bound by receptors present on the recipient cells (**b**), or fuse with the plasma membrane of these cells (**c**). Cells that receive information from glioma cells can, in turn, produce MVs, light yellow (**d**) and exosomes, dark yellow (**e**), which contain factors able to further stimulate glioma cell proliferation and invasion. In a normal astrocyte (**C**) AQP4 forms orthogonal arrays of particles (OAPs), localized in the cell endfeet (groups of small ovals drawn in red). In the glioma cell, AQP4 (red circles) is neither included in OAPs, nor localized; in addition, AQP4 levels are upregulated.

The radical changes of volume mentioned above certainly also involve water transport. Although this latter process is partially due to passive co-transport with other molecules and ions [161], transcellular water flow is mainly mediated by aquaporins (AQPs), specialized tetrameric channels, at least 13 different isoforms of which have been identified [162,163,164]. Many AQPs are also present in the central nervous system [32], the most represented of which are AQP1, AQP4 and AQP9 [32,161]. AQP1, present in the choroid plexus, seems to be involved in cerebrospinal fluid (CSF) formation [165]. AQP4 is present both in astrocytes and neurons [166], whereas no aquaporin is expressed by the brain capillary endothelial cells that constitute the anatomical basis of the blood-brain barrier (BBB) [167]. Interestingly, AQP4 in astrocytes is expressed in a polarized way: the water channels are, indeed, mainly present at the astrocytic endfeet that contact the vessels (Figure 1), both at the level of the BBB and at the CNS–CSF interface, thus suggesting a role for them in the establishment and maintenance of the BBB function [32,168]. Intriguingly, at the level of the contact points, astrocytic AQP4 is included in complexes known as orthogonal arrays of particles (OAPs), the formation of which, as well as polarity are established during development [169,170,171] and depend on both intracellular proteins (e.g., α-syntrophin) [172] and extracellular proteoglycans (e.g., agrin) [173]. Interestingly, the complexes also contain the already mentioned inwardly rectifying potassium channel Kir4.1 [167]. Finally, AQP9 is an aquaglyceroporin, probably involved also in the transport of monocarboxylates (e.g., β-hydroxybutyrate and lactate), glycerol and urea. It is present in different cell types, including astrocytes and some classes of neurons [32,174]. Many studies suggest involvement of AQPs in glioma cell ability to invade the surrounding tissue: as mentioned, indeed, penetration along the narrow extracellular spaces which surround the vessels requires cell volume changes and extracellular fluid fluxes, both largely generated by AQPs themselves [175,176]. Moreover, AQPs are probably involved in the formation of the peri-tumoral edema, which characterizes human brain cancers and affects the outcome of the pathology [177]. Actually, two kinds of tumor-associated edema are known: (i) cytotoxic, in which cells swell because of malfunctioning of the Na^+^/K^+^-ATPase, which cause Na^+^ retention, with consequent water accumulation in the intracellular fluid and (ii) vasogenic, in which breakdown of the BBB has been observed. Breakdown of the BBB is essentially due to the release by glioma cells of factors that stimulates proliferation of the brain capillary endothelial cells (BCECs). BCECs are characterized by tight junctions (TJs) that are not present in the endothelial cells that line all the other vessels in the body but, when induced to proliferate, they lose TJs and, as a consequence, BBB becomes leaky. Breakage of BBB allows extravasation of intravascular solutes and water tends to enter the brain along hydrostatic gradients, no more counteracted by opposing osmotic forces; this causes accumulation of water in the extracellular fluid, which is, indeed, the basis for edema formation [32,177]. In addition, a profound modification of AQP expression has been noticed. For example, AQP1, which is not expressed normally in endothelial cells, in brain cancer is highly expressed in BCECs and could be directly involved in vasogenic edema [165,178]. AQP4 is also upregulated in brain tumors and a clear correlation has been found between AQP4 levels and patients’ survival time. Moreover, an intracellular AQP4 redistribution has been described, which is higher in the tumor infiltration areas (Figure 1) [179,180].

### 2.5. Hypoxia, Metabolic Reprogramming and Angiogenesis

Like in most solid tumors [181], uncontrolled proliferation of glioma cells consumes oxygen supply and generates varying degrees of hypoxia, also inducing intratumoral necrosis [182,183,184,185]. Moreover, shortage of oxygen inhibits the activity of prolyl-hydroxylase domain-containing enzymes, which use molecular oxygen to hydroxylate their substrates, among which the α subunit of the hypoxia-inducible transcription factors (HIF); in normoxic conditions, hydroxylated HIFα molecules are then poly-ubiquitinated and degraded by the proteasome. When hydroxylation is inhibited, ubiquitination and degradation are inhibited as well; undegraded HIFα enters the nucleus, heterodimerizes with the HIFβ and binds to hypoxia-response elements (HREs), present in the promoters of hypoxia-regulated genes, which will be activated [186]. Among the activated genes, some encodes metabolic enzymes, such as lactate dehydrogenase A (LDHA), which can in turn induce a modification of glioma cell metabolism and behavior [187]. A generally accepted concept is that glioma cells, like other tumor cells, have enhanced glycolytic metabolism and reduced oxydative phosphorylation, even in the presence of oxygen (aerobic glycolysis); this metabolic behavior is known as Warburg effect, after the observations done by Otto Warburg in the fifties [188]. Actually, the shift toward glycolytic metabolism seems to occur also in normal proliferating cells [189] and, in cancer cells, at the earliest phases of cancerogenesis, before evident shortage of oxygen, thus suggesting that cancer (and, in general, proliferating cells) find some kind of benefit in it. Aerobic glycolysis produces piruvate that is then reduced to lactate by LDHA, at the same time allowing oxidation of NADH back to NAD+, which will be reutilized to fuel glycolysis. The lactate produced in this reaction is transported outside the cell, by monocarboxylate transporters (MCTs), together with a proton, thus causing acidification of the microenvironment. Interestingly, it has been recently reported that MCT1 expression is upregulated in hypoxic conditions [190] and that acidosis drives in turn reprogramming of the fatty acid metabolism, promoting β-oxidation, through an increase in mitochondrial proteins acetylation and deacetylation of histones [191,192]. Since lactate can also bind specific receptors in the brain [193], we cannot exclude that this molecule, once outside the cell, can also have more specific, still unknown, regulatory functions in cancer cell adaptation.

A further important aspect of metabolic reprogramming of glioma cells is upregulation of glutaminolysis [194]. This amino acid is converted by glutaminase to glutamate, which is then involved in transamination reactions, allowing production of other amino acids, on one hand and giving rise, on the other, to α-ketoglutarate (α-KG), which can enter the tricarboxilic acid (TCA) cycle. Replenishment of the TCA cycle is mainly aimed at producing citrate that, transported to the cytoplasm, will be used for lipid biogenesis [195]. Production of membrane lipids and cholesterol is indeed of the most importance for rapidly dividing cells that need to produce new membranes. Of course, rapidly dividing cells also need to synthesize nucleotides: thus, part of glucose-6-phosphate produced in the first step of glycolysis is diverted to the pentose phosphate pathway (PPP), through which it is converted to ribose-5-phosphate [192]. Moreover, the increase of the AMP/ATP ratio allows activation of the AMP-dependent serine/treonine kinase (AMPK), which increases extracellular lipid internalization and reduces energy expenditure by inhibiting de novo fatty acid (FA) synthesis [196]. All these adaptations allow tumor cells to obtain building blocks and energy for forming new organelles and for dividing. As mentioned above, however, malignant brain cancers are highly heterogeneous—actually, only rapidly dividing cells in the tumor show high activation of anabolic processes such as PPP while migrating cells do not, again suggesting a “go-or-grow” dichotomy [197].

Hypoxic conditions are also at the origin of new angiogenesis in the tumor. One of the genes activated by HIF encodes the vascular endothelial growth factor (VEGF), which is the main stimulator of endothelial cell proliferation, thus directing the growth of new vessels to the hypoxic regions [198]. However, HIF-1α and angiogenesis can be also activated in hypoxia-independent manner, by the Wnt/β-catenin target gene activation (c-Myc) [199].

As mentioned above, the tumor contains GSCs, which reside in different microenvironments with different properties, defined, respectively, perivascular, hypoxic and invasive niches [200,201]. The first two types of niches are intratumoral and contain aberrant blood vessels [202], as well as necrotic tissue, while the invasive niche is found at the interface between the tumor and the healthy brain, where GSCs adhere to the normal vessels (vascular niche) [202], inducing endothelial cells to assume an aberrant behaviour and to invade the healthy tissue [201]. In all these niches, a cross-talk between GSCs and the other cells occurs, based on production of soluble factors. At the perivascular site, for example, at least TGFβ [203] and FGF2 [204], released by endothelial cells, as well as VEGF [205], produced by GSCs, are involved. Importantly, TGFβ signaling upregulates expression of MMPs, while reducing that of MMP physiological inhibitors: TIMPs [206].

A fundamental cross-talk among vessels and GSCs also occurs at the invasive niche, where, for example, endothelial cells produce stromal-derived factor (SDF)-1 (also known as CXCL12) and bradykinin, while GSCs express the corresponding receptors (C-X-C chemokine receptor type 4, CXCR4 and bradykinin receptor 2, BR2, respectively) [201]. 

Interestingly, one aspect of intra-tumor heterogeneity is also the existence of non-mitotic territories, the genesis of which still remains to be clarified. Recently, for example, it has been reported an unexpected role of beta-catenin in determining the anti-proliferative behaviour of these territories: in particular, by stimulating the production of a microRNA (miR-302), which targets cyclin D1, beta-catenin reduces stemness properties in some tumor cells; this effect seems to be induced by a protein known as Dedicator of cytokinesis protein 4 (DOCK4) [207].

Another factor, known to stimulate both proliferation and migration, is the epidermal growth factor (EGF); indeed, overexpression of its receptor (EGFR) is a feature which characterizes high-grade gliomas, with the highest expression level at the invasive niche [6]. *EGFR* gene is amplified in 40% of the malignant gliomas and about one half of these glioma cells have a mutant form of the receptor (EGFRvIII) that lacks the ligand-binding domain, thus becoming constitutively active [6]. 

Homing at the vessels (vessel niche) and migrating along them (invasive niche), cancer cells and GSCs in particular can gain access to oxygen and nutrients that are necessary for their metabolism. However, this route also exposes invading cells to difficulties, such as the necessity to adapt to narrow spaces (see the previous section), by drastic reduction of their volumes and competition with normal astrocytes and pericytes, which stably interact with the vessels at the basal lamina, contributing to BBB formation (Figure 1). Finally, in order to migrate, cancer cells have to overcome the repulsive signals produced by the normal endothelial cells [201]. In general terms, we can conclude that cancer GSC properties are influenced by the specific microenvironments and are thus different in the different niches, which contribute to glioma cell heterogeneity.

### 2.6. Non-Coding RNAs

In the last two decades, much interest has been attracted by the existence of what has been called the dark matter of the genome, which is the existence of a large amount of DNA, which increased with evolution, which does not encode proteins but very often encodes regulatory, non-coding RNAs [208]. The most studied among non-coding RNAs are microRNAs (miRNAs), small RNAs of about 22 nucleotides, able to pair with complementary sequences, present on target RNA transcripts, called microRNA recognition elements (MREs) [209]. Pairing usually results in the target mRNA degradation or, at least, in repression of its translation. Beside miRNAs, other non-coding RNAs, longer than 200 nucleotides and therefore called “long non-coding RNAs” (lncRNAs), have been discovered. Many different functions have been attributed to lncRNAs, among which that of functioning like “sponges” for miRNAs: lncRNAs can indeed contain MREs and bind specific miRNAs, thus competing with their target mRNAs, repression of which will be correspondingly decreased [210].

Several studies have reported alteration in the concentration of specific microRNAs (miRNAs) in brain cancer. Interestingly, most (but not all) of these miRNAs have been found to have a tumor suppressor role (a few examples are reported in Table 2), since their target mRNAs encode for proteins that, when over-expressed, can be associated to cancer development, as reported in the previous sections.

Deregulated lncRNAs have been found as well; as expected, if they really have a “miRNA sponge” role, those targeting miRNAs with tumor suppressor activity have an oncogenic role, since they restrain specific miRNAs from inducing degradation of mRNAs encoding oncogenic proteins (Table 3). For example, miR-141 targets Spindle and Kinetochore Associated protein 2 (SKA2) and functions as a tumor suppressor [237]; similarly, miR-370 targets beta-catenin and cyclin E2 (CCNE2), thus acting as a tumor suppressor as well [256,257]; on the other hand, LncRNAs HOTAIR (HOX transcript antisense RNA) and KCNQ1OT1 (KCNQ1 opposite strand/antisense transcript 1) target, respectively, miR-141 [237,263,264] and miR-370 [257], acting as oncogenic factors (Table 2 and Table 3). In contrast, for example, miR-21 has an oncogenic role [220,221,222], while the LncRNA CASC2 (Cancer susceptibility candidate 2), which targets miR-21, functions as a tumor suppressor [265] (Table 2 and Table 3). Similarly, miR221/222 have an oncogenic role (by targeting TIMP2 and semaphorin 3B) [249,250,251] and the LncRNA GAS5 (Growth arrest-specific 5), which targets miR-222, functions as a tumor suppressor [263,266,267]. However, the reciprocal action of these two classes of RNAs is not always such clear and they can apparently have a synergic function. For example, miR-152-3p, which targets mRNA encoding DNA methyl transferase 1 (DNMT1) is a tumor suppressor [242], like the LncRNA called ADAMTS-AS2 (ADAM metallopeptidase with thrombospondin motif, antisense RNA 2), which also affects DNMT1 expression [265].

## 3. The Pawns of Invasion: Extracellular Vesicles (EVs)

As we have discussed in the previous sections, the ability of glioma cells to grow and invade the surrounding tissue strictly depends on a cross-talk between cancer cells and their environment. This cross-talk has been suggested to be mediated, at least in part, by molecules exchanged through extracellular vesicles (EVs) (Figure 1).

### 3.1. Extracellular Vesicles: Secretion by Producer Cells and Interaction with the Cell Environment

EVs are spheroidal membrane structures, production of which seems to be highly conserved in evolution, from bacteria [291] to human cells [210]. They have been classified into two main subgroups, depending on their origin: (i) membrane vesicles (MVs), also called ectosomes, which directly bud from the plasma membrane, with a process that resembles viral budding and (ii) exosomes, which derive from exocytosis of the so called multivesicular bodies (MVBs) [292]. In addition, a significant proportion of vesicles released from cells is given by apoptotic bodies. These different populations of vesicles have been traditionally separated on the basis of sizes and composition; many recent analyses suggest, however, which the differences are not completely clearcutting [210,293,294]. Therefore, the more general term “extracellular vesicles” (EVs) is often preferable and has been used in this review. 

It is worth noting that the term “exosome” was first used to describe a physiological process: expulsion by exocytosis, from a MVB, of unwanted or obsolete molecules (e.g., transferrin receptors), during reticulocyte maturation [295,296]. For a while, however, in the scientific literature, EVs were mentioned almost entirely in relation with tumorigenesis. Nowadays, it has been universally accepted that, thanks to their ability to transfer nucleic acids, proteins and lipids, EVs are involved in several processes, in both physiological and pathological conditions [292]. It is likely that, from an evolutionary point of view, EV production first evolved to allow discarding of unwanted/excess material. Possibly, the process also acquired with time an important adaptive function by allowing to level potentials of individual cells within a population and/or to synchronize the activities of different cell types in a tissue. The same capacity, however, can easily turn into pathology when cells excrete, via EVs, molecules that can “infect” the surrounding cells [297], or modify the extracellular environment in a way that allows spreading of the pathology (see below). We found, for example, which cultured oligodendroglioma cells discard through EVs the histone variant H1.0, which might otherwise contribute to cell differentiation [298]; on the other hand, the same EVs contain matrix metalloproteases able to digest aggrecan [299].

Although secretion of EVs from a producer cell and their interaction with the recipient cells (through specific receptors, or by a sort of endocytosis, or by fusion with the plasma membrane) have been now clearly demonstrated, the steps that determine specific sorting of molecules to nascent EVs as well as the mechanisms that allow recipient cells to accept EV-mediated signals are still matter of debate. For example, fundamental protagonists of EV-mediated intercellular exchange of information are different species of both coding and non-coding RNAs: these RNA stocks are different depending on the producing cell types and the physiological state of the cells themselves. Moreover, the EV-RNA profiles are strongly influenced by pathological conditions, such as hypoxia, oxidative stress, infections and tumorigenesis [210]. How are chosen, from time to time, these RNA species to be packaged into EVs? Probably, some sequences present in the RNA molecules and able to be specifically recognized by RNA-binding proteins (RBPs), on one hand and specific nucleotide modifications, on the other hand, are involved [210,294]. Some RBPs should be the same that in the normal brain allow subcellular prelocalization of mRNAs [300]. Now, it has been also shown that exosome biogenesis depends on the endosomal sorting complexes required for transport (ESCRT), which are responsible for most intracellular processes involving membranes. The same complexes have been also suggested to have a role in sorting RNAs to exosomes [301]. Moreover, it has been shown that many cytosolic as well as membrane-bound enzymes contain RNA-binding domains and could thus contribute to RNA sorting to EVs [210,294]. In addition, lipids can also play an important role, probably by interacting with membrane proteins in the plasma membrane microdomains from which EVs bud.

Once released from the cells, EVs may have different fates: (i) they might be recognized and bound by specific receptors on the recipient cells; it has been reported that the sites of interaction are enriched in heparin sulphate proteoglycans (HSPGs) and that binding of EVs to these molecules activates endocytosis of the particles, while also triggering a transduction pathway that involves ERK1/2 signaling [302]; (ii) they can fuse with the recipient cells; or (iii) they can break outside the cells, releasing their content into the ECM, from where they can be destroyed or/and interact with specific membrane receptors. These different events also involve RNA-protein complexes that can be simply destroyed in the ECM or reach the recipient cells [210]. Interestingly, it has been reported that, after a brief exposure to glioma EVs, brain capillary endothelial cells undergo changes in the expression of many genes, some of which do not seem related to a direct transfer of RNA into them. This finding highlights the existence of more than a single mechanism for modifying gene activity in the recipient cells [223].

Actually, EVs seem to be involved in a variety of physiological processes in the normal brain, such as glial-neuronal communications in synaptic formation, functioning and plasticity, in metabolic exchanges and so on [261,292]. As shown for many other tumors [303], however, gliomas release a much higher amount of EVs [13,212,292,304,305,306,307,308,309]. Thanks to their ability to transfer proteins, lipids and nucleic acids, EVs can affect in many ways the tumor microenvironment. In addition, whereas cancer cells are poorly able to cross the BBB, EVs do it and can be found in most body fluids, thus suggesting that they might be used as early biomarkers [12] and, perhaps, as carriers in next generation therapies.

### 3.2. How EVs Can Both Directly and Indirectly Modify the Extracellular Matrix

Tumor cells are able to modify ECM by producing EVs that contain extracellular proteins, such as the extracellular matrix protein 1 (ECM-1) [310] and Collagen IV [311], as well as ECM remodeling enzymes.

Giusti et colleagues [307] showed, for example, which vesicles produced by glioma cells contain the MMP-2 gelatinase, both in pro-enzimatic and active form, as well as pro-MMP9. These proteins are also able to form complexes in vesicles. Other proteins present in these EVs are plasminogen activators, such as PA–PAI complexes, tissue type-PA (tPA) and urokinase type-PA (uPA) [306], as well as MMP tissue inhibitors, such as TIMP1 and TIMP2, which contribute to the angiogenic activity related to tumor growth [219]. Similarly, EVs released from oligodendroglioma cells in culture contain Adamts1, Adamts4 and Adamts5 active aggrecanases and, indeed, degrade aggrecan in a dose-dependent manner [299].

Another protein, able to act as an ECM remodelling factor and present in GBM EVs, is Cathepsin D (Table 1). This latter enzyme has also an indirect role because activates other cysteine proteases. Moreover, its concentration in serum is directly related to the tumor grade [312].

Cancer cells can also cause ECM remodeling in an indirect manner: glioma cell-derived vesicles can indeed induce in vitro adjacent cells, in particular cancer-associated fibroblasts (CAF), to secrete components of the ECM [313]. EVs secreted by various cancer cells (including glioma cells) also contain tissue transglutaminase and fibronectin that are able to increase sinergistically the mitogenic activity of receiving cells, such as fibroblasts and endothelial cells; in this case, ECM proteins could help to diffuse the tumor by influencing the division rate of the other cells in the niche [314]. Interestingly, Trylcova and colleagues [315], after testing the effect of CAF conditioned media on the proliferation and chemotaxis of glioma cells in vitro, also analyzed, by immunofluorescence, glioblastoma samples from 20 patients, by using markers typical for CAFs. They revealed the regular presence in the samples of mesenchymal cells expressing CAF markers, thus indicating the potential role of CAF-like cells also in vivo [315].

Similarly, GBM-released EVs induce in GBM-associated microglia the overexpression of MT1-MMP, thus further supporting tumor growth [316]. Moreover, the presence of Semaphorin3A (Sema3A) at EV surface, causes anomalous cell-substrate adhesion and the loss of the endothelial barrier integrity [317].

Interestingly, exosomes released under hypoxic conditions are enriched in metalloproteinases and lysyl oxidases and can thus promote angiogenesis [318].

SPARC (secreted protein acidic and rich in cysteine) is a protein strongly expressed in perivascular cells, adjacent to GBM vessels [319]; it modulates the interactions between cells and the extracellular matrix and promotes migration and invasion. Recently, this protein has been also found in glioma-associated vesicles [310].

One of the main proteins of EVs, β1-Integrin (ITGB1), was also recently found in vesicles produced by GBM. This protein is thought to have not only a structural role but also the ability to interact with β5-Integrin (ITGB5); the resulting complexes, bound to fibronectin, may stimulate invadopodia formation [310].

It is important to remind that composition and concentration of the EVs released into the extracellular environment as well as their capturing depend on the actual conditions of the producing/receiving cells. Treatment of gliomas with drugs or ionizing radiation can alter their production and/or capturing. Arscott et colleagues showed, for example, which radiations induce an increase of exosome release in a dose- and time-dependent manner. Moreover, these exosomes were found to enhance cell motility, by activating members of the focal adhesion kinase (FAK) signaling cascade [320].

Furthermore, recent studies demonstrated that vesicles produced by irradiated glioma cells (ionizing radiation) were able to modulate MMP2 activity in recipient cells, not by direct transfer but by regulating the expression of the corresponding gene [321].

### 3.3. EVs as Inducers of Gene Expression Modifications

Extracellular vesicles can also contain signaling proteins. Recently, for example, active K-Ras has been found in exosomes released by glioblastomas. Interestingly, it has been demonstrated that its sorting to vesicles requires farnesylation, thus demonstrating the importance for sorting of protein interaction with membranes. In the same paper, it has been also shown that Ras present in the vesicles can be experimentally coprecipitated with some proteins which are normally part of the already mentioned endosomal sorting complex required for transport (ESCRT) [322]. ESCRT is required for many activities involving modification of the plasma membrane (PM), such as viral budding and formation of the multivesicular body (MVB), from which a specific class of EVs (exosomes) derives. Beside four main proteins (ESCRT 0, I, II and III), it contains also accessory factors and some of these components (ESCRT III, in particular) are fundamental for inducing the membrane curvature necessary for forming exosomes inside the MVB [323].

We can hypothesize that, like farnesylated Ras, many other transforming proteins can access EVs because of their interaction with proteins and/or lipids of the PM, possibly through ESCRT. 

Under the same hypothesis, given the interaction between the PM and the cytoskeleton, it is likely that also cytoskeletal components can access EVs. For example, in a study involving EVs secreted by six different glioblastoma cell lines, it has been found that they contain high levels of proteins correlated with invadopodia formation, among which Arp3. Beside these proteins, the authors found integrin-β1, insulin-like growth factor 2 receptor and programmed cell death 6-interacting protein [310]. On the other hand, membrane proteins can reach the vesicles in an even simpler way; for example, the already mentioned CLIC1 channel, so important for modulating the ability of the cells to change their volume, has been also found in EVs [157].

Some of the proteins present in EVs more strictly correlate with the hypoxic state of the glioma cells; among these proteins, in addition to those, already mentioned, which are involved in ECM remodeling and angiogenesis, caveolin (CAV), lysyl oxidase and interleukin 8 (IL8) have been also found [318]. Interestingly, it has been recently shown that EV internalization into recipient cells is inhibited by siRNA-mediated knockdown of caveolin-1, flotillin-1, RhoA, Rac1 and PAK1 but not clathrin heavy chain, thus suggesting that EVs enter cells predominantly via clathrin-independent endocytosis and macropinocytosis [324].

In Section 2.6 we discussed the potential role of non-coding RNAs in brain cancers but we now know that non-coding RNAs, as well as mRNAs, can also exist outside the cell, mostly complexed with RNA-binding proteins and very often associated with EVs. EV-transferred RNAs can be captured by surrounding cells and induce profound modifications in gene expression of the recipient cells: (i) mRNAs can be translated; (ii) miRNAs can target the endogenous mRNAs; (iii) lncRNAs can function as guiding and/or scaffolding elements for chromosomal organizing- and transcriptional-factors [325,326]; moreover, they can act as sponges for endogenous miRNAs, thus reducing their ability to target endogeneous mRNAs [327]. In other words, all RNA species can act as epigenetic determinants, able to change gene expression in recipient cells [210]. Interestingly, by taking advantage of glioma cell capacity to produce miRNA-containing exosomes, Fareh et al. [328] have obtained primary glioma cells that stably produces miR-302-367. They found that these cells package into exosomes a high amount of miR-302-367, which are then internalized by the surrounding cells. Most important, these miRNAs were then able to inhibit expression of their targets (among which cyclins D and A and E2F1), thus efficiently reducing tumor development [328].

Glioma cell-derived mRNAs that accumulate into EVs form a highly complex population which includes a variety of transcripts driving proliferation, immune suppression and tissue invasion [219]; although these mRNAs are representative of the entire transcriptome of glioma cells, as discussed in Section 3.1, some species are clearly enriched in EVs [329], thus suggesting the existence of specific sorting mechanisms, which might be based on the interaction of mRNAs with different classes of RNA-binding domains, which could be present on proteins with other, better known, functions [300].

As in the case of mRNAs, also the population of miRNAs present in EVs is representative of the species most expressed in the glioma cells from which EVs originate. For example, miR-21 and miR-26a oncogenic miRNAs were both abundantly found in glioma cells and in glioma cell-derived EVs [224]. In some cases, however, it seems that sorting events localize specific miRNAs to the vesicles [223]. Again, as in the case of mRNAs, we can hypothesize that specific RBPs are involved in the process. It has also been found that microRNAs enriched in EVs show post-transcriptional modifications, such as uridylated 3′ end; for example, mature miR-451, one of the most actively secreted by glioblastoma, contain two U residues at the 3′-end [13]. Thus, it is likely that RBPs, which specifically recognize the U residues, can be involved in sorting.

Recently it has been also demonstrated that glioma-cell derived EVs also contain lncRNAs, such as linc-POU3F3 [279]. In particular, Lang and coll. demonstrated that EV-transported linc-POU3F3 can be internalized by microvascular endothelial cells, where it causes an increase of the gene and protein expression levels of bFGF, bFGFR and VEGFA, thus setting the conditions for angiogenesis [279].

The ability of EVs to transport proteins as well as coding and noncoding RNAs poses, however, a fundamental problem concerning the real capacity of all these EV-carried molecules to be internalized by the surrounding cells at levels that can significantly modify their activities. It is indeed important to be reminded that, as suggested by Chevillet et al., most EVs might contain less than one molecule of a given miRNA [330]. However, at least in some cases, transfer of molecules has been clearly demonstrated. For example, glioblastoma-released EVs can actively transfer miR-21 and miR-451 to microglia and macrophages, where they target c-Myc mRNA [331]. Actually, it had been already suggested that the cells of the monocytic lineage, including monocytes, macrophages and microglia, were particularly affected by glioma cells; these effects, (namely increased cytokine secretion, increased phagocytic capacity of macrophages and increased expression of MT1-MMP by microglial cells) were mediated by EVs of glioblastoma origin but not by EVs of non-glioblastoma origin [316].

A final comment concerns the fact that many RNA-binding proteins are also able to bind DNA and can thus modify gene expression, once arrived in the recipient cells; in other words, such proteins might travel to the EVs because of their interactions with RNAs of different classes but then rely on their DNA-binding activity to transform cell behavior [210,332].

## 4. Conclusions

The ability of cancer cells to invade the healthy brain tissue is a pathologic property of gliomas that contributes to the failure of the therapies currently adopted for the patients and essentially based on surgery, followed by radiotherapy and/or chemotherapy. A further obstacle derives from development of drug resistance. In the last decade, more and more aspects of brain cancer biology have been discovered, highlighting the molecular alterations that accompany cell transformation that generate an invasive phenotype. To understand the cellular and molecular bases of these events is of the most importance in order to envisage new approaches to therapy.

Among the intriguing aspects of invasion is also to consider the fact that glioma cells, like probably all the other cancer cells, secrete a variety of molecules by releasing into their environment extracellular vesicles. It has been clearly demonstrated that EVs are involved in several events which promote cancer development, such as: suppression of the immune response, stimulation of cancer growth, angiogenesis and invasion. Interestingly, it has been also found that, although glioma cells are not able to cross the blood-brain barrier, EVs can, at least in part, do it and can be detected in the peripheral blood. This finding could offer a fundamental tool for rapid and non-invasive diagnosis. Moreover, a few laboratories are already working on the possibility of using EVs for therapeutic aims.

However, a few questions, concerning biogenesis and general function of EVs, are still open. For example, it is not yet clear which are the mechanisms that activate EV release and why, indeed, cancer cells present with a much higher production of them. On the other hand, it is not completely clear how the surrounding cells are induced to catch the vesicles.

Given the cross-talk existing among the cancer cells and the surrounding cells (namely, normal brain-, immune- and endothelial-cells), it is clear that to clarify the above-mentioned points can be of great help for a better understanding of the cancer ecosystem and, possibly, for setting new approaches in the therapy of malignant brain tumors, which are, up to now, still almost incurable.

## Figures and Tables

**Table 1:** Examples of extracellular matrix ECM components and ECM modifying enzymes that have been reported to be up/down-regulated in gliomas. In the last column, a few examples of therapies targeting these proteins are given.

Factor	Function	Up/Down Regulated in Glioma [References]	Therapies Targeting These Proteins [References]
ADAMs 8, 9, 10, 17, 19	extracellular disintegrin and metalloproteases	up-regulated [39]	ADAM 10 and 17 [40]
ADAM-22	inhibitor of astrocyte proliferation	downregulated in high-grade gliomas [41]	over-expression of miR-145 targets, among other genes, also ADAM-22 [42]
ADAMTS-4 and ADAMTS-5	degrade lectican and small leucine-rich repeat families of proteoglycans	expression correlates with glioma invasiveness [43]	no example of specific targeting found
Cathepsin B and D	extracellular proteases	upregulated in high-grade gliomas [44,45,46,47]	tivozanib diminished glioblastoma multiforme (GBM) cell invasion by impairing the proteolytic cascade of cathepsin B/urokinase-type plasminogen activator (uPA)/matrix metalloproteinase-2 (MMP-2) [48]
CCN1	heparin-binding protein; interacts with the integrins α-v β-3 and α-6β-1 and increases the migration of glioma cells	highly up-regulated in primary gliomas and invasive glioblastoma cell lines [49]	potential therapy based on oncolytic HSV1 (OV) [50]
Collagen Type IV	the major structural component of basement membranes	up-regulated [51]	the lysyl oxidase inhibitor β-aminopropionitrile disrupts collagen structure in the tumor and inhibits tumor angiogenesis and glioblastoma multiforme growth in a mouse orthotopic brain tumor model [52]
Hyaluronan	the major component of the brain ECM	up-regulated in primary brain tumors [53] It stimulates secretion of matrix metalloproteases	hyaluronidase can improve penetration of therapeutic agents into brain tumors [54]
Matrix metalloproteinase (MMP)-1	interstitial collagenase	expression increases with WHO grade [55]	a collection of new drugs targeting matrix metalloproteases have been tested in vitro. Among them: 2-Amino-2-[2-(4-octylphenyl)]-1,3-propanediol hydrochloride (FTY720) [56], chlorotoxin [57], ellagic acid [58], fucoxanthin [59], caffeic acid [60]. Moreover, silencing of specific genes appears as a promising tool for inhibiting growth and invasiveness of glioma cells, by reducing expression of matrix metalloproteases [61,62]
MMP-2	gelatinase activity	highly upregulated, secreted, activated [63,64,65]	
MMP-3	broad substrate specifity	highly upregulated, secreted, activated [66]	
MMP-7	broad substrate specifity	highly upregulated, secreted, activated [67]	
MMP-9	gelatinase activity	highly upregulated, secreted, activated [64,65]	
MMP-11	does not degrade laminin, fibronectin and elastin; has a strong activity on serine protease inhibitor α1-antitrypsin and insulin-like growth factor binding protein-1 (IGFBP-1)	expression increases with WHO grade [55]	
MMP-12	degrades soluble and insoluble elastin, type IV collagen, fibronectin, fibrillin-1, laminin, vitronectin, chondroitin sulfate and heparin sulfate proteoglycans, MMP2/3 activation	elevation of MMP-12 by tenascin-C in glioma [68]	
MMP-19	degrades various ECM components including collagen type IV, nidogen-1, fibronectin, tenascin-C isoform, aggrecan and laminin-5-gamma-2-chain	expression increases with WHO grade [55]	
MMP-26	degrades type IV collagen, fibronectin, vitronectin, alpha 1-antitrypsin (A1AT), insulin-like growth factor-binding protein 1 (IGFBP) and activates MMP9	significantly up-regulated [69]	
(MT1)-MMP/MMP-14	involved in the maturation of active MMP-2	highly upregulated, secreted, activated [70]	no example of specific targeting found
Tenascin-C	plays a crucial role in angiogenesis, proliferation and cell migration	up-regulated [71,72]	a peptide that bound to tenascin C has been isolated by phage display peptide library. The selected peptide specifically recognized tenascin C protein in xenograft mouse tissue [73]
Tenascin-R	influences cell adhesion, neural cell migration, cell-matrix interaction and axon outgrowth	increasingly down-regulated with glioma progression: (in grade IV glioblastoma only a weak TN-R expression is detected [72]	no example of specific targeting found
TIMP-1	natural inhibitor of MMPs	higher levels in GBM compared to lower grade glioma [74]	2-Amino-2-[2-(4-octylphenyl)]-1,3-propanediol hydrochloride (FTY720) [56]
Thrombospondin 1 (TSP-1)	Implicated in cancer cell, adhesion, migration, invasion, inhibition of angiogenesis	may decrease with tumor grade [75]	no example of specific induction found

**Table 2:** Involvement of microRNAs (miRNAs) in glioma growth and invasion: putative mode of action of a few miRNAs, with some of their suggested targets. In the last column, when available, references in which the presence in EVs of these miRNAs has been discussed.

miRNA	Proposed Mode of Action	Some Proposed Targets [References]	Presence in EVs [References]
miR-1	tumor suppressor	Annexin A2 [211]	[10,212]
miR-7	tumor suppressor	EGFR, FAK, IRS1/2 [213,214]	-
miR-9	oncogenic	Stathmin [81]	Found in EVs from breast cancer cell lines [215]
miR-10b	oncogenic	UPAR, RhoC [216]	[10]
miR-16	tumor suppressor	BCL2, WIP1-ATM-p53 pathway [217,218]	[219]
miR-21	oncogenic	TIMP3, RECK4, PDCD4, β-catenin [220,221,222]	[13,212,223]
miR-26a	oncogenic	PTEN, Rb, MEKK2 [224]	[212]
miR-26b	tumor suppressor	BCL2 [225]	[212]
miR-29	tumor suppressor	DNMT3A and 3B. [226]	-
miR-29a	oncogenic	PTEN [223]	[223]
miR-30e	oncogenic	NFkB, VEGF-C, MMPs [223]	[223]
miR-34a	tumor suppressor	PKCε, PD-L1 [227,228]	-
miR-93	oncogenic	Integrin β8 [229,230]	[212]
miR-98	tumor suppressor	IKK-ε [231]	-
miR-124	tumor suppressor	AURKA, Smad4 [232,233]	[224]
miR-128	tumor suppressor	EGFR, PDGFRA, EphB2, p70S6K PRC1, PRC2 (reduces levels of phospho-Akt and derepresses p21 expression) [224,234]	[10,13,224]
miR-130b	oncogenic	CYLD [235]	Found in EVs from prostate cancers [236]
miR-141	tumor suppressor	SKA2 [237]	-
miR-142	tumor suppressor	Rac1 [238]	-
miR-146b	tumor suppressor	MMPs [239,240]	[10,241]
miR-152-3p	tumor suppressor	DNMT1 [242]	-
miR-181	tumor suppressor	Bcl-2, KPNA4 [243,244]	-
miR-200c	tumor suppressor	EGFR, AKT [245]	-
miR-210	oncogenic	Glycerol-3-phosphate dehydrogenase 1-like; increased levels of HIF3A and of VEGF [13]	[13]
miR-218	tumor suppressor	IKK-β, Bmi1, RTK-HIF pathway [246,247,248]	-
miR-221/222	oncogenic	TIMP2, SEMA3B [249,250,251]	[13,223]
miR-296	oncogenic	HGS, STAT5A [252,253]	-
miR-320	oncogenic		[241]
miR-326	tumor suppressor	SMO, Notch2, NOB1 [254,255]	-
miR-370	tumor suppressor	beta-catenin, CCNE2 [256,257]	-
miR-451	tumor suppressor	Akt1, CyclinD1, MMP-2, MMP-9 and Bcl-2, LKB1 [258,259,260]	[13,261]
miR-592	tumor suppressor	IGFBP2 [262]	-
miR-5096	oncogenic	Kir4.1 [160]	[223]

**Table 3:** Involvement of long non-coding RNAs (LncRNAs) in glioma growth and invasion: putative mode of action of a few LncRNAs, with some of their suggested targets.

LncRNA	Proposed Mode of Action	Some Proposed Targets
ADAMTS-AS2	tumor suppressor	DNMT1 [265]
CASC 2	tumor suppressor	miR-21 [268]
CRNDE	oncogenic	miR-186, miR-384/PIWIL4 [269,270]
GAS 5	tumor suppressor	miR-196a, miR-222 [263,266,267]
H19	oncogenic (generates miR-675)	Cadherin 13 (CDH13) [271,272,273]
HOTAIR	oncogenic	PDCD4, miR-141, SNORD47 [237,263,264,274]
HOTTIP	oncogenic	miR-101 [275]
HULC	oncogenic	ESM-1; PI3K/AKT/mTOR [276]
KCNQ1OT1	oncogenic	miR-370 [257]
LINC0000125	oncogenic	miR-4775 [277]
LINC-POU3F3	oncogenic	POU3F3; bFGF, bFGFR, VEGFA [278,279]
LINK-A	oncogenic	LDH-A [280]
MALAT-1(NEAT-2)	oncogenic	miR-101 [281]
PLAC 2	tumor suppressor	ribosomal protein (RP)L36, STAT1 [282]
TUG1	tumor suppressor	miR-26a, miR-144, miR-299 [283,284,285]
UCA1	oncogenic	miR-122 [286,287]
XIST	oncogenic	miR-29c, miR-137, miR-152 [288,289,290]

## Abbreviations

ADAM17	ADAM (a disintegrin and metalloprotease domain) metallopeptidase domain 17
ADAMTS-AS2	ADAM metallopeptidase with thrombospondin motif, antisense RNA 2
ADD3	Adducin 3
AKT1	Ak strain transforming (also known as protein-chinasi B o PKB) 1
ASLNC	Anti-sense long non-coding RNA
ATM	Ataxia telangiectasia mutated
AURKA	Aurora kinase A
BCL-2	B-cell lymphoma 2
Bmi1	B cell-specific Moloney murine leukemia virus integration site 1 (Polycomb complex protein BMI-1)
CASC 2	Cancer susceptibility candidate 2
CDC42	Cell division control protein 42 homolog
CCNE1/CCNE2	Cyclin E1/Ciclina E2
CRNDE	Colorectal neoplasia differentially expressed
CYLD	Cylindromatosis (turban tumor syndrome)
DIXDC1	Dixin; DIX domain-containing protein 1
DNMT1	DNA methyl transferase 1
EGFR	Epidermal growth factor receptor
ESM-1	Endothelial cell specific molecule 1
FAK	Focal adhesion kinase
Fer1L4	Fer (Feline Encephalitis Virus-Related) Kinase-1 Like Family Member 4 (pseudogene)
FoxM1	Forkhead box M1
GAS 5	Growth arrest-specific 5
HGS	Hepatocyte growth factor-regulated tyrosine kinase substrate
IGF	Insulin-like growth factor
IGFBP	Insulin-like growth factor-binding protein
IKK	IκB kinase
IRS 1/2	Insulin Receptor Substrate 1/2
HIF	Hypoxia-inducible factor
HOTAIR	HOX transcript antisense RNA
HOTTIP	HOXA transcript at the distal tip
HULC	Highly up-regulated in liver cancer
JAG-1	Jagged-1
KCNQ1OT1	KCNQ1 (potassium voltage-gated channel subfamily Q member 1) opposite strand/antisense transcript 1 (non-protein coding)
Kir 4.1	Inward-rectifier potassium ion channel 4.1
KPNA4	Karyopherin (Importin) Subunit Alpha 4
LDH-A	Lactate dehydrogenase A
LINC	Long intergenic non-coding RNA
LINK-A	Long intergenic non-coding RNA for kinase activation
LKB1	Liver Kinase B1 (also known as Serine/Threonine Kinase 11—STK11)
MALAT-1	Metastasis associated lung adenocarcinoma transcript 1
MBD2	Methyl-CpG binding domain protein 2
MEKK2	Mitogen-Activated Protein Kinase Kinase Kinase 2
MMP	Matrix metalloproteinase
mTOR	Mammalian target of rapamycin
NEAT	Nuclear enriched abundant transcript
NEDD9	Neural precursor cell expressed developmentally down-regulated protein 9
NOB1	Nin1 (One) Binding protein 1
Notch2	Neurogenic locus notch homolog protein 2
PAK4	P21 (RAC1) Activated Kinase 4
PBX3	Pre-B-cell leukemia transcription factor 3
PDCD4	Programmed cell death protein 4
PD-L1	Programmed death-ligand 1
PI3K	Phosphoinositide 3-kinase
PIWIL	Piwi-like RNA-mediated gene silencing
PKC	Protein kinase C
POU3F3	POU Class 3 Homeobox 3
PRC 1/2	Polycomb repressor complex 1/2
PLAC 2	Placenta specific 2
PTEN	Phosphatase and tensin homolog
Rac1	Ras-related C3 botulinum toxin substrate 1,
RECK	Reversion-Inducing Cysteine-Rich Protein With Kazal Motifs
RhoC	Ras homolog gene family, member C
ROCK1	Rho-associated, coiled-coil-containing protein kinase 1
RTK	Receptor tyrosine kinase
SEMA3B	Semaphorin 3B
SIRT6	Sirtuin 6
SKA2	Spindle and Kinetochore Associated protein 2
SMAD	Small mother against decapentaplegic
SMO	Smoothened, Frizzled Class Receptor
SNORD47	Small Nucleolar RNA, C/D Box 47
Sox7/Sox9	SRY-Box 7/SRY-Box 9
STAT1/STAT5A	Signal transducer and activator of transcription 1/5A
TIMP-1/TIMP2	Tissue inhibitor of metalloproteinase 1/2
TSP 1	Thrombospondin 1
TSHZ3	Teashirt zinc finger homeobox 3
TUG1	Taurine up-regulated gene
UCA1	Urothelial cancer associated 1 (non-protein coding)
UPAR	Urokinase receptor
WIP	WAS/WASL interacting protein
Wnt	Wingless-related integration site
XIST	X-chromosome inactive specific transcript
YAP1	Yes associated protein 1
